# Indirect risk effects reduce feeding efficiency of ducks during spring

**DOI:** 10.1002/ece3.3714

**Published:** 2017-12-12

**Authors:** Adam C. Behney, Ryan O'Shaughnessy, Michael W. Eichholz, Joshua D. Stafford

**Affiliations:** ^1^ Department of Zoology Center for Ecology Cooperative Wildlife Research Laboratory Southern Illinois University Carbondale IL USA; ^2^ Frank C. Bellrose Waterfowl Research Center Illinois Natural History Survey Institute of Natural Resource Sustainability University of Illinois Havana IL USA; ^3^ Present address: Avian Research Section Colorado Parks and Wildlife Fort Collins CO USA; ^4^ Present address: Borderlands Research Institute Sul Ross State University Alpine TX USA; ^5^ Present address: Department of Natural Resource Management U.S. Geological Survey South Dakota Cooperative Fish & Wildlife Research Unit South Dakota State University Brookings SD USA

**Keywords:** foraging, nonlethal effects, perceived predation risk, risk‐taking, waterfowl

## Abstract

Indirect risk effects of predators on prey behavior can have more of an impact on prey populations than direct consumptive effects. Predation risk can elicit more vigilance behavior in prey, reducing the amount of time available for other activities, such as foraging, which could potentially reduce foraging efficiency. Understanding the conditions associated with predation risk and the specific effects predation risk have on prey behavior is important because it has direct influences on the profitability of food items found under various conditions and states of the forager. The goals of this study were to assess how ducks perceived predation risk in various habitat types and how strongly perceived risk versus energetic demand affected foraging behavior. We manipulated food abundance in different wetland types in Illinois, USA to reduce confounding between food abundance and vegetation structure. We conducted focal‐animal behavioral samples on five duck species in treatment and control plots and used generalized linear mixed‐effects models to compare the effects of vegetation structure versus other factors on the intensity with which ducks fed and the duration of feeding stints. Mallards fed more intensively and, along with blue‐winged teal, used longer feeding stints in open habitats, consistent with the hypothesis that limited visibility was perceived to have a greater predation risk than unlimited visibility. The species temporally nearest to nesting, wood ducks, were willing to take more risks for a greater food reward, consistent with an increase in a marginal value of energy as they approached nesting. Our results indicate that some duck species value energy differently based on the surrounding vegetation structure and density. Furthermore, increases in the marginal value of energy can be more influential than perceived risk in shaping foraging behavior patterns. Based on these findings, we conclude that the value of various food items is not solely determined by energy contained in the item but by conditions in which it is found and the state of the forager.

## 1 INTRODUCTION

Indirect risk effects by predators on prey can have as much or more of an impact on prey populations than direct consumptive effects (Creel & Christianson, 2008; Pangle, Peacor, & Johannsson, 2007; Preisser, Bolnick, & Benard, 2005). Predation risk has been repeatedly shown to affect the behavior of prey (Lima, 1998; Werner & Peacor, 2003) including changes in movements/habitat selection (Gilliam & Fraser, 1987; Gordon, Feit, Gruber, & Letnic, 2015; Kotler, Brown, & Hasson, 1991), group size (Creel, Schuette, & Christianson, 2014), vigilance (Creel et al., 2014), and/or foraging behavior (Guillemain et al., 2007; Kotler et al., 1991), among others (described in Lima, 1998 and Peckarsky et al., 2008). Predation risk has been shown to limit individuals’ foraging effort/intake rate in a wide range of taxa (Bednekoff, 2007; Verdolin, 2006) and one way animals mediate risk while foraging (Bednekoff, 2007; Lima & Bednekoff, 1999; Lindstrom, 1989; Verdolin, 2006) is through the use of vigilance behavior (Beauchamp, 2015b). However, vigilance can reduce intake rate (Fritz, Guillemain, & Durant, 2002), leading to a tradeoff between food intake and safety while foraging. Because fecundity of animals is often limited by nutrient acquisition (Ankney & MacInnes, 1978; Milenkaya, Catlin, Legge, & Walters, 2015), predation risk that diminishes the efficiency with which animals acquire nutrients can reduce foragers’ fecundity.

Animals assess predation risk through direct interactions with predators (Greig‐Smith, 1982; Marzluff, 1988; Morosinotto, Thomson, & Korpimaki, 2010) or indirectly through inter‐ or intraspecific communication (Citta & Lindberg, 2007; Danchin, Boulinier, & Massot, 1998; Emmering & Schmidt, 2011; Zanette, White, Allen, & Clinchy, 2011), indices of predator abundance (Eichholz, Dassow, Stafford, & Weatherhead, 2012; Forsman, Monkkonen, Korpimaki, & Thomson, 2012), or perceived ability to detect and escape predators (Metcalfe, 1984) or hide from them (Lima, 1987). For terrestrial systems, Verdolin (2006) found in a meta‐analysis that habitat characteristics produced consistently stronger effects than odor or live predators as a correlate of predation risk. From an evolutionary perspective, it seems likely that animals have evolved the ability to rapidly assess predation risk from their surroundings rather than relying on continuous assessment of direct or indirect predator observations. Therefore, some metric describing the vegetation structure (i.e., cover) in the immediate area where individuals are foraging is commonly used to represent risk (Beauchamp & Ruxton, 2016; Lima, 1987; Poysa, 1994; Slotow & Rothstein, 1995; Spencer, Crowther, & Dickman, 2014).

The relationship between cover and risk may be positive if cover limits the ability of prey to detect approaching predators (Whitfield, 2003) or negative if cover provides a safe place for animals to escape predators or camouflage prey from predators (Lazarus & Symonds, 1992). For example, white‐crowned sparrows (*Zonotrichia leucophrys*) feeding farther from cover had a lower vigilance rate (Slotow & Rothstein, 1995). In arctic ground squirrels, Wheeler and Hik (2014) attributed reduced foraging efficiency and greater giving up densities in shrub‐dominated areas to lower visibility and a greater perceived predation risk. Similarly, Beauchamp (2015a) found that when semipalmated sandpiper's (*Calidris pusilla*) visibility was obstructed, they used a higher level of vigilance. Alternatively, the buffy‐headed marmoset (*Callithrix flaviceps*) used vigilance more when under less extensive leaf cover (Ferrari & Ferrari, 1990). There is also evidence that intermediate levels of cover can result in increased or decreased perceived predation risk. Townsend's ground squirrels used vigilance more often when in medium height vegetation than in low or tall vegetation (Sharpe & Van Horne, 1998). Alternatively, Poysa (1994) found in teal (*Anas crecca*) that vigilance stints were shortest when individuals were at intermediate distances to cover. Similarly, gerbils perceived short cover as a safe refugia, whereas tall cover and completely open were both perceived as more risky (Embar, Kotler, & Mukherjee, 2011).

In addition to cover, food density has been shown to affect vigilance levels. McNamara and Houston (1994) showed theoretically that an increase in resource abundance (magnitude of food reward) should increase the level of risk animals are willing to endure. Beauchamp (2014) found that semipalmated sandpipers used less vigilance when food density was greater. Similarly, Pays et al. (2012) found that impalas used vigilance less frequently when in enriched food patches. They found an interaction between visibility and patch enrichment on vigilance, suggesting that when visibility was good, resource quality was the primary factor influencing vigilance, whereas when visibility was poor, predation risk was the main driver of vigilance. Additionally, minnows refused to feed in a patch where they had recently encountered a simulated predator unless food density was substantially greater than the other patches (Pitcher, Lang, & Turner, 1988). Repasky (1996) also found a negative relationship between foraging patch profitability and vigilance in sage sparrows (*Amphispiza belli*) and black‐throated sparrows (*A. bilineata*). Furthermore, for many animals, food availability and cover are positively correlated (Naylor, Eadie, Smith, Eichholz, & Gray, 2005), creating additional complexity to the risk vs. reward tradeoff if cover is perceived as risky.

Factors other than cover and food density have also been proposed to explain how animals perceive/manage predation risk while foraging. Aggregating has been repeatedly shown through theory and empirical studies to reduce individuals’ predation risk (Caraco, 1979a,b; Powell, 1974) through dilution of risk among individuals and/or many eyes searching for predators (Dehn, 1990). Furthermore, individuals may coordinate their vigilance so that one individual scans for predators while others feed (Brandl & Bellwood, 2015). The reduced predation risk associated with group foraging not only allows individuals to feed more intensively, but may also facilitate sampling of adjacent patches resulting in more accurate information on the relative foraging profitability of those patches (Pitcher & Magurran, 1983). An individuals’ marginal value of energy has also been proposed to affect how much risk animals are willing to take. Individuals that greatly value energy (either through high demand or low reserves) should be willing to risk more (Brown, 1999); a starving animal takes more risks than an animal with lots of energy reserves (Lima, 1988).

Foraging animals typically alternate between stints of actively feeding and nonfeeding (e.g., vigilance). We define a “feeding stint” as the time period an individual has its head‐down searching for or procuring food items (predator detection is generally diminished during this time) and “foraging” as a general state encompassing both feeding stints and other behaviors (e.g., vigilance or transition periods) between feeding stints. We refer to the proportion of behavioral observations for which an animal was in a feeding stint during foraging as “feeding intensity.” Using longer feeding stints likely results in greater risk because foragers are going for longer periods without scanning, reducing their chances of detecting approaching predators before they get too close to escape. Therefore, predation pressure probably limits the duration of feeding stints (i.e., the amount of time a foraging individual goes without scanning; e.g., Poysa, 1988; Randler, 2006).

Spring migratory ducks represent an interesting opportunity to evaluate indirect risk effects while foraging. A variety of duck species forage in close proximity using foraging methods associated with variable risks (e.g., dabbling on the surface with only their head submerged and diving with their entire body submerged) and forage in different habitats with substantial variation in vegetation structure and density. Spring is an extremely important time period for the acquisition of nutrient reserves for use during reproduction, representing a time of high energy and nutrient demand (Arzel, Elmberg, & Guillemain, 2006). During late winter and spring at midlatitudes, many ducks consume primarily seeds to build lipid reserves, whereas invertebrates make up a minor diet component (Hitchcock, 2009; Tidwell, Webb, Vrtiska, & Bishop, 2013). Vegetation density in wetlands is typically correlated with duck food abundance (Naylor et al., 2005; Straub et al., 2012) because vegetation produces the seeds and provides food and cover for invertebrates.

Research relating to feeding and vigilance behavior is traditionally posed in a more basic ecology context. However, they can also be used to address management strategies. For example, substantial resources are devoted to conservation planning and habitat management/acquisition for nonbreeding ducks (North American Waterfowl Management Plan Committee 2012). Habitat conservation planners commonly use daily ration bioenergetics models to guide habitat conservation for nonbreeding ducks (Playa Lakes Joint Venture 2008; Soulliere et al., 2007). Most of these models assume that all energy is equally accessible and valuable to ducks no matter where or under what conditions the energy is found. However, under certain conditions, predation risk may limit the efficiency with which ducks consume energy, thus reducing its value. For example, if predation risk necessitates more vigilance while ducks feed in areas with dense cover, those ducks’ intake rate will be lower, necessitating longer overall feeding times or less overall consumed food. The value of energy found under these conditions could be reduced in biological planning models compared with energy found under conditions more conducive to efficient exploitation (lower predation risk). There has been very little research on what factors influence the feeding efficiency of ducks. The studies that have been completed suggest that shallow feeding is perceived as less risky for dabbling ducks (Guillemain, Duncan, & Fritz, 2001; Guillemain, Fritz, & Blais, 2000; Poysa, 1987a) as well as feeding at intermediate distances to cover and in larger flocks (Poysa, 1994).

We tested for an indirect impact of predation on duck foraging behavior by assessing the relative support for risk effects versus other factors such as energetic demand in shaping foraging behavior of ducks. We reduced confounding between cover and food availability by manipulating food abundance in wetlands with different structural characteristics. Our first objective was to assess how vegetation density (an index of predation risk) and food availability altered overall feeding intensity of ducks compared with other factors such as energetic demand and flock size. Under this hypothesis, individuals should feed more intensively when they perceived a lower predation risk (Brown & Kotler, 2004). Secondly, we assessed whether feeding stint durations were related to the vegetation structure or food density. We tested these hypotheses using five duck species that differ in life history characteristics: blue‐winged teal (*Anas discors*), mallard (*Anas platyrhynchos*), wood duck (*Aix sponsa*), lesser scaup (*Aythya affinis*), and ring‐necked duck (*Aythya collaris*).

Mallards, blue‐winged teal, lesser scaup, and ring‐necked ducks commonly roost in relatively open habitats. Therefore, we predicted that those species would perceive cover as more risky and alter their feeding behavior accordingly (lower intensity, shorter stints) when foraging near cover (Table 1). Wood ducks typically inhabit more vegetated wetlands (forested, dense emergent vegetation); therefore, we predicted that wood ducks would perceive open habitats as more risky (Table 1). Furthermore, wood ducks were much nearer to nesting than the other species and thus, females likely have a greater marginal value of energy as they build nutrient reserves for use during nesting. Therefore, we predicted that factors related to energetic demand (i.e., date or sex) or food availability would be more influential in shaping wood duck feeding behavior than in other species (Table 1).

**Table 1:** Predictions regarding factors influencing feeding intensity of ducks during spring migration

Species	Perceived risk (greater vegetation density)	Energetic demand
Blue‐winged teal	More risk—lower feeding intensity	No effect
Mallard	More risk—lower feeding intensity	No effect
Lesser scaup	More risk—lower feeding intensity	No effect
Ring‐necked duck	More risk—lower feeding intensity	No effect
Wood duck	Less risk—greater feeding intensity	Females—increasing with date

## 2 MATERIALS AND METHODS

### 2.1 Study area

We conducted this research in the Wabash River region of Eastern Illinois. We used two sites about 116 km apart: one in Lawrence County, Illinois and the other in Gallatin and White Counties, Illinois. Land‐cover in the Wabash River region was historically dominated by forest but was cleared for row‐crop agriculture, which is the dominant land‐use currently in the region. We selected wetlands that were large enough to encompass two 0.4 ha identical sample plots that were relatively homogeneous within and between the plots and would remain inundated for a 3‐week period. We used three broad wetland types in this study: forested, emergent, and open water. Forested wetlands contained mature trees and a canopy over the wetland and consisted of species like maple (*Acer* spp.) and oak (*Quercus* spp). Emergent wetlands had much vegetation emerging through the water surface characterized by sedges (*Cyperus* spp.), millets (*Echinochloa* spp.), smartweeds (*Polygonum* spp.), and other grasses and forbs. Open water wetlands had very little or no vegetation emerging through the water surface. Based on 25–30 random sample locations within each plot, mean water depth ± SE of emergent, forested, and open water wetlands was 41.1 ± 4.6, 42.1 ± 5.3, and 49.4 ± 3.0 cm, respectively.

Potential predators of ducks in our study area are mammals (coyote, bobcat, foxes, river otter, and raccoon) and raptors (large suite of hawks, eagles, and owls). Little information exists on nonhunting cause‐specific mortality of ducks during the nonbreeding season (especially spring migration). In the Mississippi alluvial valley, nine of 11 mortalities, for which the cause was identified, were from avian predators (Davis, Afton, & Cox, 2011). We speculate that in our study area during migration, avian predators were the primary threats because ducks were feeding in water, in which mammals probably lack the speed and stealth necessary to capture a duck. Furthermore, most mammalian predators are nocturnal and we conducted our observations during the day.

### 2.2 Experimental design

This study occurred during late winter and early spring, 2012 and 2013. We established two adjacent 0.4 ha plots and placed a wooden stake every 21 m around each border, so observers could identify the plot boundary. The plots were separated by a minimum of 25 m and we refer to the paired plots as a block. We attempted to establish three blocks in each wetland type each year at each site but did not achieve this every year due to lack of availability of inundated wetlands. In one randomly selected plot (treatment plot) in each block, we spread 816 kg (2018 kg/ha) of corn (*Zea mays*) kernels by hand with buckets or using a seed spreader attached to a boat. The adjacent control plot did not receive any corn. Treatment plots received corn treatments once each year, and the treatments were staggered throughout the study period to ensure some plots of each wetland type had recently been treated throughout the study period. We selected corn because many ducks are primarily consuming seeds to build lipid reserves during this time period and at this latitude (Hitchcock, 2009; Tidwell et al., 2013) and corn is an energy‐rich item (Kaminski, Davis, Essig, Gerard, & Reinecke, 2003), which we predicted ducks would select.

### 2.3 Field methods

We randomly selected the order of blocks and plots for observations each week. Observations were conducted on each block for 3 weeks following treatment. Where possible, we observed both plots in a block from the same observation point simultaneously but where this was not possible due to visual obstruction from vegetation, we observed from two different observation points. Observation points were within 40 m of a plot border, and we attempted to place observation points the same distance from each plot in a block to reduce any bias based on distance to observer. We observed plots during morning (0.5 hrs before sunrise to 2.5 hrs after sunrise) and evening (2.5 hrs before sunset to 0.5 hrs after sunset) observation periods. We placed a treestand, tripod stand, or ground blind at each observation point to reduce any effect of observers on ducks and to increase visibility of plots to observers. Six observers collected behavioral data each year, and they were rotated through blocks to reduce any potential bias associated with individual observer. Observers arrived at observation points at least 0.5 hrs prior to observation periods to reduce any disturbance effect.

We used instantaneous focal‐animal sampling to construct time‐budgets of ducks (Altmann, 1974). Instantaneous samples consisted of recording a randomly selected duck's behavior every 20 s, for 5 min, and occurred every 15 min on each plot being observed. Behaviors were recorded as one of two categories: feeding (feeding on surface, feeding under water surface, feeding by up‐ending, or feeding by diving) and nonfeeding (resting, alert, agonistic, courtship, self‐maintenance, and swimming; Poysa, 1983; Lovvorn, 1989).

We selected five focal species upon which to focus our sampling: mallard, blue‐winged teal, lesser scaup, wood duck, and ring‐necked duck. A randomly ordered list of the species of interest and sex was generated each week to facilitate selection of an individual for focal sampling. A random number table was generated prior to observations to facilitate selection of an individual by counting from the left or right (randomly chosen) the random number of individuals of the species and sex of interest. This was repeated for one individual of each species and sex of interest present in the plot with about 15 min between samples. After acquiring one sample of each species of interest, we went back to the first species and worked through the list of species again. Although we used a random selection protocol to select individuals on which to conduct behavior samples, ducks were not marked, therefore, it is possible that the same individual may have been sampled multiple times. However, this is unlikely because generally ducks were in large flocks (mean = 64 individuals) so the probability of sampling the same bird twice, using our random selection protocol, was low. Following each focal‐animal sample, we noted the location of the duck on a map of the plot and flock size of which the focal‐animal was a part. We defined a flock as a group of ducks with no more than 10 m spacing between nearest neighbors and included ducks of any species because the effect of group size on vigilance is not limited to single species groups (Metcalfe, 1984). Flocks regularly spilled outside of experimental study plots and we included individuals outside of plots in the flock size count if they fit our definition of being in the same flock as the focal individual. After each observation period, we went to focal‐duck locations and measured water depth and visibility using a 1.5 m Robel pole marked in 10‐cm intervals. We noted the number of 10‐cm intervals that were completely visible from a distance of 10 m from four directions. The four visibility measurements were averaged for each location.

In addition to the direct behavioral observations, we used a video camera (Sony Handycam HDR‐CX190) to record other ducks for up to 10 min and made sure to zoom out to allow estimation of flock size. We used this additional sampling method to facilitate accurate estimation of feeding stint durations. We watched the videos on a computer and constructed continuous focal‐animal samples using the Jwatcher program (Blumstein, 2006). As a duck transitioned to a new behavior, we keyed in the appropriate behavior and the program constructed the time‐budget with starting times of each behavior which allowed us to calculate feeding stint duration (seconds).

To assess whether ducks were actually consuming the added corn, we collected ducks with a shotgun. We collected ducks opportunistically while conducting behavioral observations when a duck got within about 30 m of the blind and we had observed it feeding. Directly following collection, we injected a 10% buffered formalin solution into the esophagus to prevent post‐mortem digestion (Swanson & Bartonek, 1970) and placed a zip‐tie at the base of the skull to keep esophageal contents from spilling out (Hitchcock, 2009). After collection, no more behavioral data were collected during that observation period. Collected ducks were frozen and transported to a laboratory facility at Southern Illinois University where the digestive system was examined for the presence or absence of corn kernels.

### 2.4 Statistical analyses

#### 2.4.1 Feeding intensity

To assess factors that influenced feeding intensity for each species, we used feeding vs. not feeding as a binary response variable in generalized linear mixed models (package lme4 in R; Bates, Maechler, & Bolker, 2013; R Core Team, 2013) with a binomial error distribution and logit link function. Random effects included sampled duck (multiple observations on the same individual), nested within block (paired control/treatment plots). We used a multistage modeling strategy in which we first found the most parsimonious model incorporating variables we classified as background or nuisance variables: year, site, and water depth. We included water depth at this first step because it could be considered both a risk variable (deeper feeding is more risky) or an energetic demand variable (deeper foraging requires more energy). Then, we added variables representing perceived predation risk and energetic demand to this baseline model. Our predation risk fixed effects included visibility (Robel pole reading), wetland type, and flock size. Energetic demand fixed effects included date and sex. We compared models from each category to each other to assess the relative importance of predation risk vs. energetic demand in shaping feeding behavior of ducks. We added the variable treatment in additive and interactive relationships to the best models in each group to assess how the relationship between risk (proportion of time feeding) and reward (food treatment) was influenced by the variables that may alter ducks’ perceived predation risk or energetic demand. We included a null model incorporating only random effects in all analyses as a general indicator of candidate model performance (Burnham & Anderson, 2002).

#### 2.4.2 Stint duration

To assess our predictions regarding the effects of visibility, flock size, and food availability on feeding stint duration, we used linear mixed‐effects models (package lme4 [Bates et al., 2013] in R [R Core Team, 2013]) with stint duration as the response variable (log transformed to reduce heteroscedasticity) in an analysis similar to that for feeding intensity.

#### 2.4.3 Model inference

For each analysis, we confirmed that models were consistent with their assumptions by examining residual plots as described in Zuur, Ieno, Walker, Saveliev, and Smith (2009). We evaluated all models using an Information Theoretic approach with Akaike's Information Criterion (AIC_c_) and model weights (*w*_*i*_; Burnham & Anderson, 2002). To graphically show variable effects, we generated predictions by varying the effect(s) of interest across its range while holding other variables in the model constant at their mean. All predictions are presented on the response scale (probability of feeding).

## 3 RESULTS

We established and sampled 25 blocks distributed among emergent (*n* = 11), forested (*n* = 7), and open water (*n* = 7) wetlands between 15 February and 11 April, 2012 and 2013. We conducted 1,310 instantaneous focal‐animal samples on wood duck (*n* = 434), mallard (*n* = 331), blue‐winged teal (*n* = 267), ring‐necked duck (*n* = 228), and lesser scaup (*n* = 50). The average length of focal‐animal samples was 4.3 min (range = 1.7–5.0 min). Overall, feeding and vigilant behaviors dominated activity budgets (86% of observations). Using the video‐recorded data, we conducted 251 continuous focal‐animal samples on mallard (*n* = 80), blue‐winged teal (*n* = 51), lesser scaup (*n* = 40), ring‐necked duck (*n* = 40), and wood duck (*n* = 40). Of the ducks collected, 44.0% of blue‐winged teal (*n* = 25), all mallards (*n* = 5), 66.7% of wood ducks (*n* = 3), 50.0% of lesser scaup (*n* = 4), and 53.3% of ring‐necked ducks (*n* = 15) contained corn. Although we did not record data on noncorn food in digestive tracts, anecdotally, it appeared that most of the ducks that did not contain corn were void of any food items.

### 3.1 Feeding intensity

**Figure 1:**
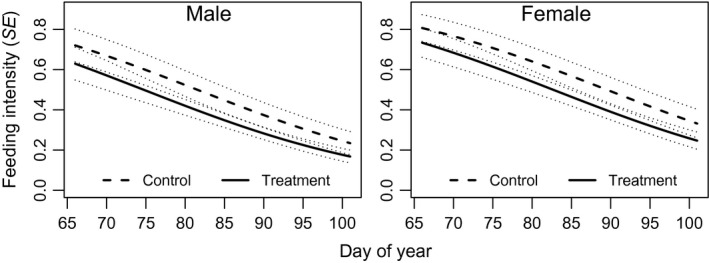
Blue‐winged teal model predicted feeding intensity (probability of an observation to be classified as feeding) at various dates for each sex and treatment level (from model ‘date + sex + treatment’). Gray dotted lines represent predicted values plus or minus one standard error. Day 65 and 100 correspond with 6‐Mar and 10‐Apr, respectively

**Figure 2:**
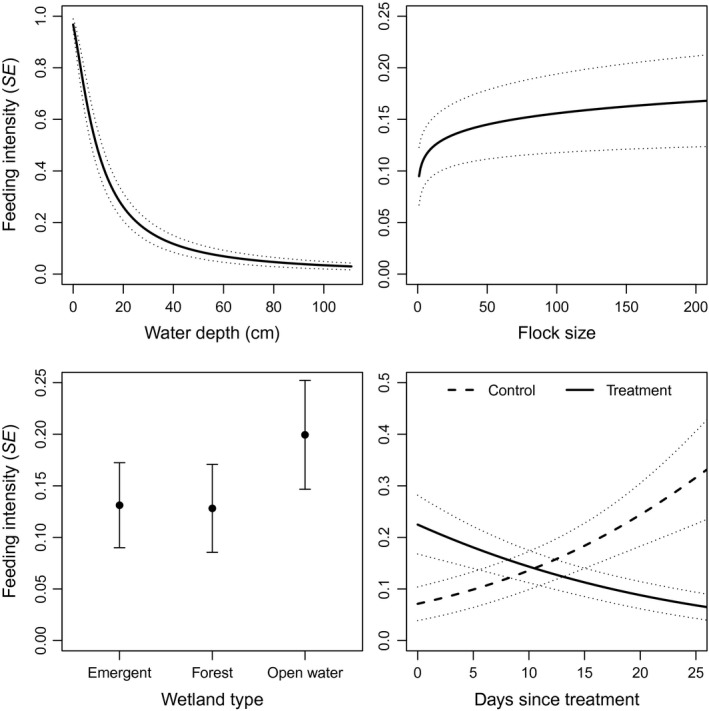
Model predicted feeding intensity for mallards at various water depths (topleft), flock sizes (topright), wetland types (bottomleft), and in treatment and control plots following treatment (bottomright). Error bars and gray dotted lines represent predicted values plus or minus one standard error. Water depth, flock size, and treatment * days since treatment plots were created from model ‘Water depth + Flock size + Treatment * Days since treatment’. The wetland type plot was created from the model ‘Water depth + Flock size + Wetland type + Treatment * Days since treatment’. In all plots, effects of interest were allowed to vary while other variables were held constant at their mean

**Figure 3:**
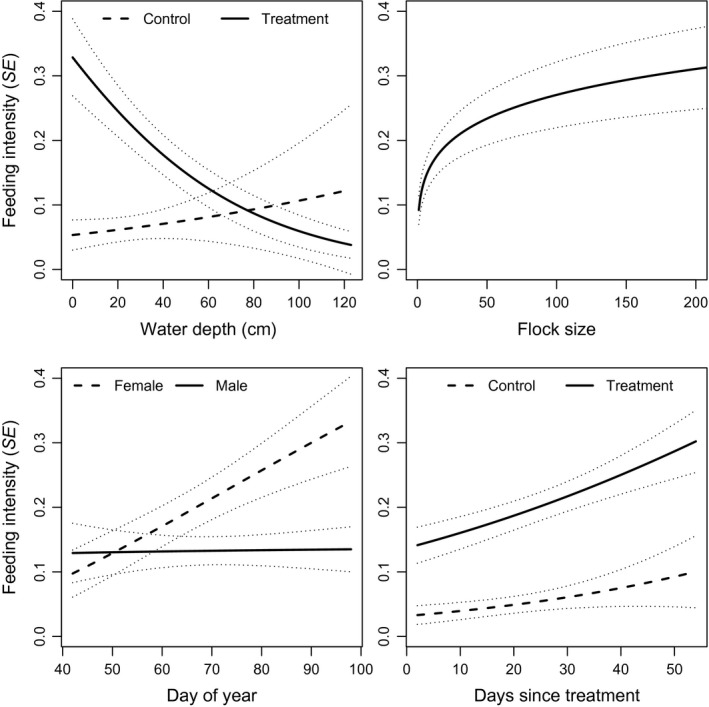
Model predicted feeding intensity ± standard error for wood ducks at various water depths in treatment and control plots (topleft), flock sizes (topright), dates and sex (bottomleft), and days as treatment in treatment and control plots (bottomright). The water depth * treatment and flock size plot were created from model ‘Water depth * Treatment + Flock size’. The date * sex plot was created from model ‘Water depth * Treatment + Date * Sex’. The treatment * days since treatment plot was created from model ‘Water depth + Flock size + Treatment * Days since treatment’. In all plots, effects of interest were allowed to vary while other variables were held constant at their mean

**Figure 4:**
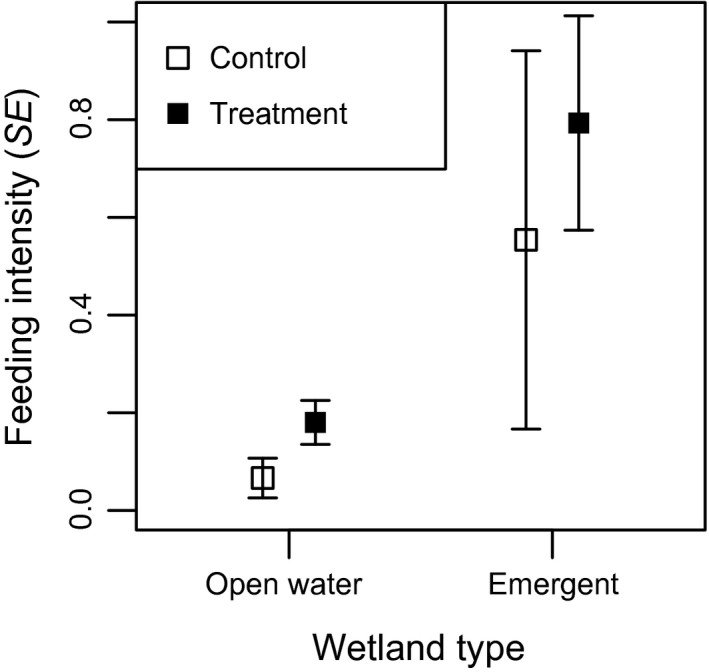
Model predicted feeding intensity ± standard error for lesser scaup in different wetland types and treatments (from model “wet type + treat”)

**Figure 5:**
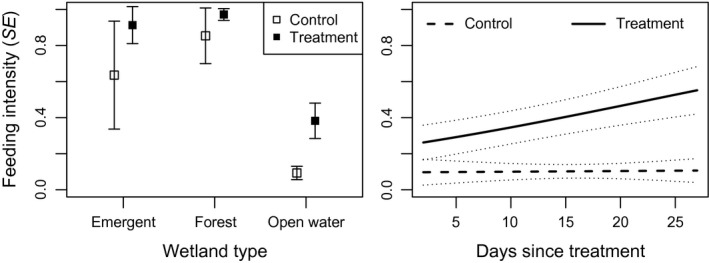
Model predicted feeding intensity ± standard error for ring‐necked ducks different wetland types and treatments (left; from model “wet type + treat”) and at various days as treatment in treatment and control plots (right; from model ‘Wetland type + Treatment * Days since treatment, holding wetland type constant to open water)

For blue‐winged teal, models representing predation risk hypotheses to explain feeding behavior were not competitive with energetic demand models (Table 2; best predation risk model: ‘Flock size’; ΔAIC_c_ = 14.9, *w*_*i*_ = 0.0). Feeding intensity decreased with date (*β* = −0.06 ± 0.01[SE]), females devoted more time to feeding than males (*β*_male_ = −0.48 ± 0.20), and feeding intensity was greater in control plots (*β*_treatment_ = −0.42 ± 0.25; Figure 1). For mallards, predation risk models outperformed other models (Table 2). Feeding intensity was much greater at shallow depths (*β* = −1.46 ± 0.21), in open water wetlands (*β*_open_ = 0.50 ± 0.30), and slightly greater in larger flocks (*β* = 0.12 ± 0.07; Figure 2). Feeding intensity increased with days as treatment in control plots but decreased slightly in treatment plots (*β*_treat*days_ = −0.13 ± 0.03; Figure 2). The best model representing energetic demand hypotheses included effects of sex and treatment and was 2.6 AIC_c_ units behind the top model. Wood duck feeding intensity was influenced by variables representing both predation risk and energetic demand (Table 2). Feeding intensity increased with flock size (*β* = 0.28 ± 0.07), and at shallow depths, wood ducks devoted substantially more time to feeding when in treatment plots as opposed to control plots (*β*_depth*treat_ = −0.77 ± 0.34; Figure 3). Females increased their feeding intensity with date, whereas males did not (*β*_male*date_ = −1.75 ± 0.96; Figure 3). Although the interaction between treatment and days as treatment appeared in a competitive model, the effect was very weak (*β*_treat*days_ = 0.00 ± 0.02; Figure 3). For lesser scaup, feeding intensity appeared to be more influenced by predation risk variables (Table 2). Feeding intensity was greater in treatment plots than control plots (*β*_treat_ = 1.13 ± 0.68) and in emergent as opposed to open water wetlands (*β*_open_ = −2.86 ± 1.33; Figure 4). There was also some support for feeding intensity increasing when visibility was less (*β* = −1.12 ± 0.65; Figure 4). The best model representing energetic demand included date and was 2.4 AIC_c_ units below the top model. For ring‐necked ducks, models representing predation risk hypotheses to explain foraging behavior outperformed those representing energetic demand (Table 2). Ring‐necked ducks fed more intensively in emergent and forested wetlands (*β*_forest_ = 1.21 ± 0.64) than open water wetlands (*β*_open_ = −2.83 ± 1.23) and when in treatment plots (*β*_treat_ = 1.80 ± 0.32; Figure 5). Although the interaction between treatment and days as treatment appeared in a competitive model, the effect was weak (*β*_treat*days_ = 0.05 ± 0.05; Figure 5). The best energetic demand model included date, sex, and treatment and was 4.8 AIC_c_ units behind the top model.

**Table 2:** Model selection results for predicting feeding intensity for blue‐winged teal, mallards, wood ducks, lesser scaup, and ring‐necked ducks. Only models with ∆AIC_c_ < 2 and null models are shown. Models were generalized linear mixed models and all models include random intercepts for block and individual duck. Models incorporating interactions also include main effects of both interacting variables

Species	Model	*K* a	∆AIC_c_	*w*_*i*_ b
Blue‐winged teal^c^	Sex + Date + Treatment	8	0.0	0.3
	Sex + Date	7	0.6	0.2
	Null	3	13.3	0.0
Mallard^d^	Water depth + Flock size + Treatment * Days since treatment	9	0.0	0.3
	Water depth + Treatment * Days since treatment	8	0.8	0.2
	Water depth + Flock size + Wetland type + Treatment * Days since treatment	11	1.3	0.2
	Null	3	58.6	0
Wood duck	Water depth * Treatment + Flock size	7	0.0	0.3
	Water depth * Treatment + Date * Sex	9	1.5	0.1
	Water depth + Flock size + Treatment * Days since treatment	8	1.7	0.1
	Null	3	55.1	0.0
Lesser scaup^d^	Wetland type + Treatment	6	0.0	0.2
	Wetland type	5	0.3	0.2
	Visibility + Treatment	6	1.6	0.1
	Visibility	5	1.8	0.1
	Treatment	5	1.9	0.1
	Null	3	3.2	0.0
Ring‐necked duck^d^	Wetland type + Treatment	7	0.0	0.3
	Wetland type + Treatment * Days since treatment	9	0.8	0.2
	Wetland type * Treatment	9	1.3	0.2
	Null	3	71.6	0.0

a Number of model parameters.

b Model weight.

c All models include additive effects of site and year except the null model.

d All models include additive effects of site except the null model.

### 3.2 Stint duration

Due to limited samples, we were not able to evaluate the effects of wetland type and treatment on feeding stint duration for each species. For blue‐winged teal, we were not able to include the effects of wetland type and treatment in the same model due to insufficient samples in both control and treatment plots. The most parsimonious model included additive effects of wetland type and behavior type (Table 3). Stint durations were longest in open water (1.3 ± 0.3 s for surface feeding) followed by emergent (0.6 ± 0.3 s), and forested (0.5 ± 0.2 s) wetlands. We also found support for a treatment effect (control = 1.3 ± 0.2 s, treatment = 0.6 ± 0.2 s for surface feeding) and weak support for a positive flock size effect (β = 0.001 ± 0.005). Mallards were the only species for which we were able to evaluate all effects of interest, although we were only able to use forest and open water wetlands. The most parsimonious model of mallard feeding stint duration included additive effects of wetland type, flock size, and behavior type (Table 3). Feeding stint duration was greater in open water (1.2 ± 0.2 s for mean flock size) than forested wetlands (0.8 ± 0.2 s) and stint duration increased weakly with flock size (β = 0.0008 ± 0.0005). Although treatment appeared in a competitive model, the effect was weak of mallards using longer feeding stints when in control plots (1.2 ± 0.2 s, holding other variables constant) compared with treatment plots (1.1 ± 0.2 s). For ring‐necked ducks, we were only able to test the effects of flock size and treatment due to lack of samples in each wetland type. Furthermore, we did not include feeding behavior because we only observed ring‐necked ducks diving. The only model to outperform the null model included flock size (Table 3; β = 0.02 ± 0.01).

**Table 3:** Model selection results for predicting feeding stint duration for blue‐winged teal, mallard, and ring‐necked duck. Models were generalized linear mixed models and all models for blue‐winged teal and mallards include random intercepts for block and individual duck. Ring‐necked duck models only included individual duck as a random effect

Species	Model	*K* a	∆AIC_c_	*w*_*i*_ b
Blue‐winged teal^c^				
	Wetland type	8	0.0	0.3
	Wetland type + Flock size	9	0.4	0.3
	Treatment	7	0.5	0.3
	Null	4	56.6	0.0
Mallard^c^				
	Flock size + Wetland type	8	0.0	0.3
	Wetland type	7	0.2	0.3
	Flock size + Wetland type + Treatment	9	0.9	0.2
	Wetland type + Treatment	8	1.8	0.1
	Null	4	51.8	0.0
Ring‐necked duck				
	Flock size	4	0.0	0.5
	Null	3	1.2	0.3

a Number of model parameters.

b Model weight.

c Feeding behavior type was included in every model except the null model.

## 4 DISCUSSION

Gaining a better understanding of indirect risk effects can help answer questions about why certain habitats are selected over others and possible consequences of those habitat selection choices on individuals and ultimately, populations. We used food manipulations to reduce confounding between vegetation structure and food abundance to examine how ducks perceive habitat characteristics in terms of predation risk versus other factors. The two response variables we chose to examine were the prevalence of active head‐down feeding (feeding intensity) and feeding stint duration. For mallards and blue‐winged teal, we found evidence consistent with the hypothesis that limited visibility due to vegetation above the water's surface obstructing their view was perceived as risky and resulted in ducks altering their foraging behavior to mediate this risk, with associated foraging efficiency costs.

Habitat characteristics (wetland type or visibility) appeared in competitive feeding intensity models for three of the five species and in two of the three species used in the feeding stint duration analysis. Although wetland type only appeared in the third most parsimonious model (ΔAIC_c_ = 1.3), consistent with our predictions, mallards tended to feed more intensively when in open water wetlands (high visibility) indicating they perceived those areas as safer. Furthermore, mallards and blue‐winged teal used shorter feeding stints when in areas with more cover. Early detection of approaching predators greatly reduces the risk of prey being killed (Lima, 1987). There is a “critical time” or “critical distance” before which a prey must detect a predator and take some evasive action to facilitate escape (Beauchamp & Ruxton, 2016; Lima, 1987; Poysa, 1987a). Visibility likely plays a key role in this critical time during which prey must detect predators to avoid being killed (Whitfield, 2003). If foragers have poor visibility (e.g., blocked by vegetation), the amount of time that a predator may be detectable before it gets to the critical time or distance may be less than when visibility is greater, creating a more dangerous or risky situation for the forager (Whitfield, 2003). It appears that when mallards’ or blue‐winged teals’ visibility was restricted, they devoted more time to predator detection with associated reductions in feeding intensity and/or feeding stint duration, presumably reducing intake rate.

Lesser scaup and ring‐necked duck behavior was also influenced by vegetation structure (wetland type); however, they fed more intensively when in areas with limited visibility. These results are interesting given that diving ducks typically use more open water habitat (Anteau & Afton, 2009; O'Shaughnessy, 2014). For lesser scaup at least, we found more model selection uncertainty and weaker effects when modeling feeding intensity, likely due to a small sample size (*n* = 50) as evidence by more models appearing competitive and the null model was not substantially worse than the top model (ΔAICc = 3.2). Therefore, we are hesitant to draw strong inference from the lesser scaup models. However, we did have a sufficient sample for ring‐necked ducks and it appears that they were willing to feed more intensively in areas with more cover, which is counter to our prediction.

Wood ducks were the only species for which we found results consistent with the hypothesis that energetic demand influences foraging behavior, although the support for energetic demand models was somewhat weak (ΔAICc = 1.5). Wood ducks were least influenced by visibility and wood duck females tended to be willing to risk the most for a greater reward and as the spring progressed, devoted more time to feeding. Wood ducks were the earliest nesting focal species and we observed nest searching behavior during this study. Wood ducks use stored lipids for egg production, which they consume and store prior to laying, whereas most protein in eggs comes directly from dietary sources (Drobney, 1980). Our results are consistent with female wood ducks taking more risks to consume and store lipids when they have arrived or are close to arrival in their breeding areas, prior to laying. Unexpectedly, blue‐winged teal devoted less time to feeding, later in the spring. This is inconsistent with an increase in energy demand for reproduction. We do not have a good explanation for this phenomenon but speculate that more first year or poor quality birds were present on our sites later in the spring, which may be less invested in reproduction and therefore, forage less. Nest success is commonly found to decline throughout the nesting period (Haffele, Eichholz, & Dixon, 2013), and this may be due to poor quality individuals arriving and initiating nesting later (Hepp & Kennamer, 1993), which is consistent with our observations for blue‐winged teal. Additionally, temperatures were warmer later in the spring which may reduce energy demand for thermoregulatory costs.

Water depth was important in predicting feeding intensity of wood ducks and mallards. Deeper foraging likely incurs a greater predation risk because a duck with its head or body underwater is not able to detect and evade an approaching predator as quickly as a duck foraging in shallower water with eyes above the surface (Poysa, 1987a). Generally, ducks feeding deeper devote less overall time to feeding and more to vigilance (Guillemain et al., 2001), suggesting that shallow foraging is not as mutually exclusive with vigilance as deep foraging and ducks perceive greater risk during stints of deep foraging. Furthermore, Guillemain et al. (2000) found that given the choice of shallow or deep water, mallards preferred shallow foraging even when food abundance was substantially greater in the deeper areas. Consistent with these studies, we found that wood ducks and mallards reduced the time they spent feeding when in deeper water. For wood ducks, the effect of water depth interacted with treatment; at shallow depths wood ducks fed much more intensively when in treatment plots. We interpret these results to mean shallow water depths enabled wood ducks to better take advantage of the superabundant food in our treatment plots, whereas in deeper water, it was not worthwhile to feed more intensively for the extra food reward.

Intake rate increases with food abundance, at least to some asymptote (Arzel et al., 2007; Fritz, Durant, & Guillemain, 2001). Therefore, ducks’ intake rate in treatment plots was likely greater (or at least equal) than in control plots when feeding with the same intensity. McNamara and Houston (1994) suggested that an increase in resource abundance (magnitude of food reward) should increase the level of risk animals are willing to endure. In our experiment, this would be realized by ducks feeding more intensively or using longer feeding stints in treatment plots. Alternatively, because intake rate increases with food density, ducks may use an increase in food density to maintain the same intake rate while reducing their risk by feeding less intensively or using shorter feeding stints. Wood ducks, lesser scaup, and ring‐necked ducks were clearly willing to risk more for a greater food reward. When in shallow water, where most feeding occurred, wood ducks were willing to devote over three times as much effort to feeding when in treatment plots compared with control plots. Alternatively, blue‐winged teal fed more intensively and used longer feeding stints when in control plots, consistent with the hypothesis that increased food density enabled them to reduce their risk by feeding less intensively. Rather than risk more for a greater food reward in treatment plots, blue‐winged teal reduced their predation risk in areas with more food while presumably maintaining at least the same intake rate as in control plots. This result is more consistent with the idea that predation risk drives behavior more than food abundance.

Interestingly, flock size was only important in predicting feeding intensity for wood ducks and mallards; however, it was important in predicting feeding stint duration for all three species we were able to include in the analysis. While there is much theoretical support for a group size effect (Pulliam, 1973), the empirical support is more variable (Beauchamp, 2008; Elgar, 1989; Poysa, 1987b). The time‐budget benefits of adding group members declines as group size increases (Beauchamp, 2010; McNamara & Houston, 1992). Under most conditions, the added behavioral benefits of increasing group size begin to decrease at around five individuals (inferred from figures 1, 2, 3, 4, and 6 in McNamara & Houston, 1992). The average flock size in this study was 64 individuals (range: 1–3,204). We may not have observed a flock size effect in all species because the majority of the flock sizes we observed were larger than that at which there are any additional benefits to animals’ time‐budgets.

In conclusion, the assumption of most biological habitat conservation planning models (Playa Lakes Joint Venture 2008; Soulliere et al., 2007) that a unit of energy is equally valuable to ducks across the landscape under all conditions, appears to be violated. We show that risk effects limit mallard and blue‐winged teals’ abilities to feed efficiently when they are in areas with limited visibility, which reduces the value of energy available to them in these types of wetlands. In other words, our results indicate that the variation in the energetic value of a food item to a duck is not only determined by the metabolizable energy of the food item, but also by the risk that the forager perceives and the way that perceived risk affects foragers feeding efficiency. The next step is to conduct detailed intake rate experiments to determine exactly how much intake rates are reduced due to perceived risk in various habitats. These estimates could then be used to penalize the contribution of energy from risky habitats toward the overall energy availability on the landscape.

## AUTHORS’ CONTRIBUTIONS

ACB, RO, MWE, and JDS conceived and designed the experiments. ACB and RO performed the experiments. ACB analyzed the data and wrote the manuscript. Other authors provided editorial advice.

## DATA ACCESSIBILITY

We will archive data with the Dryad Digital Repository.

## CONFLICT OF INTEREST

None declared.
